# Inhalational versus intravenous anesthetic for cerebrovascular accident outcomes after surgical revascularization for adult moyamoya disease

**DOI:** 10.1186/s12871-025-02958-7

**Published:** 2025-02-15

**Authors:** Yifei Cheng, Chaochao Zha, Xuehua Che, Yingwei Wang

**Affiliations:** Department of Anesthesiology, Huashan Hospital, Fudan University, 12 Middle Wulumuqi Road, Shanghai, 200040 People’s Republic of China

**Keywords:** Moyamoya disease, Surgical therapy, Anesthesia, Sevoflurane, Propofol

## Abstract

**Purpose:**

To compare the effects of inhalational anesthetics and intravenous anesthetics on the neurological function of patients with moyamoya disease (MMD) after vascular bypass surgery.

**Methods:**

The clinical anesthesia data of patients were retrospectively collected. Patients who underwent bypass grafts with general anesthesia from January 1st, 2019, to December 31st, 2020, in Huashan Hospital affiliated with Fudan University, were selected. The primary endpoint was stroke incidence within seven days after anesthesia, and the secondary endpoints included transient neurological deficits (TNDs) and incidence of postoperative Epilepsy.

**Results:**

We compared the data of MMD patients who received inhalational anesthetics (Sevoflurane anesthetics, *n* = 197, group S) and intravenous anesthetics (Propofol anesthetics, *n* = 219, group P). The stroke incidence in the two groups (group S vs. group P) was 6.6% vs. 5.9% (OR = 0.893; 95% CI, 0.404–1.976; *p* = 0.780), and the group S vs. group P of TNDs incidence was 32.5% vs. 31.1% (OR = 0.936; 95% CI, 0.619-0.1.415, *p* = 0.753). At discharge, anesthetics didn’t affect the neurological endpoint. Intravenous anesthetics provided patients with better hemodynamics compared with inhalational anesthetics during MMD vascular bypass surgery (group S vs. group P, ARV_SBP_: 6.4 vs. 5.2, *p* < 0.001, ARV_DBP_: 3.9 vs. 3.3, *p* = 0.002, ARV_MBP_: 4.5 vs. 3.8, *p* = 0.001,). There were statistical no differences in the NHISS score (S group vs. P group = 2:1, *p* = 0.082) at 7 days after surgery, but mRS score (S group vs. P group = 2:1, *p* < 0.001) at 7 days after surgery, as well as the mRS score at 6 months of follow-up (S group vs. P group = 0:0, *p* < 0.001), although the difference in scores was small.

**Conclusion:**

Our data indicated that both inhalational and intravenous anesthetics had protective effects on patients who underwent MMD bypass grafts. MMD patients who received inhalational anesthetics and intravenous anesthetics had similar odds of neurological deficits. When comparing long-term clinical data, most patients experience good neurological recovery after receiving inhalation or intravenous anesthesia, when compared p_75_ mRS score(S group vs. P group = 3:1)in 6 month indicate that intravenous anesthetics might be more suitable for patients undergoing MMD bypass grafts. During the operation hemodynamic stability in the propofol group is greater than that in the sevoflurane anesthesia group.

## Background

Moyamoya Disease (MMD) is a chronic cerebrovascular disease with unclear etiology and is also called spontaneous basilar artery occlusion or skull base vascular abnormalities [1]. The significant features of this disease are thickened intima, gradual arterial lumen narrowing to occlusion, and compensatory enlargement of perforator arteries. MMD patients usually have symptoms such as reduced cerebral perfusion pressure and inadequate collateral blood supply, leading to reduced blood flow at distal regions [2]. Conservative treatment has poor efficacy on MMD patients, and surgical revascularization under general anesthesia is the main treatment method. Currently, commonly used general anesthesia drugs include intravenous anesthetics and inhalation anesthetics, which have different mechanisms for brain protection. Propofol, a commonly used intravenous anesthetic, has been demonstrated in animal experiments can reduce cerebral ischemia-reperfusion injury by regulating neurotransmitters and other mechanisms [3], while sevoflurane, a commonly used inhalation anesthetic, can exert brain protection by reducing oxidative stress and inflammation [4]. As there is a controversy of research on the effects of these two types of drugs on patients undergoing revascularization surgery for MMD patients, we hypothesize that in adult patients with Moyamoya disease undergoing surgical revascularization, inhalational anesthesia is associated with better cerebrovascular outcomes, such as a lower incidence of postoperative cerebrovascular accidents, compared to intravenous anesthesia, we aims to investigate the effects of representative drugs of these two anesthetics (propofol vs. sevoflurane) on perioperative complications and postoperative neurological function in patients with MMD.

## Subjects and methods

### Research subjects

The participants included in this study were from a single center retrospective study. The clinical data of 461 MMD patients (age 18–69 years old) who underwent intra- and extracranial revascularization from January 1st, 2019, to December 31st, 2020, were retrospectively analyzed. This study was approved by the ethics committee of the Huashan Hospital affiliated with Fudan University (Ethics number KY2024-795). Patients and their family members were informed of relevant research content, and informed consent was signed. Inclusion criteria: (a) patients were diagnosed with MMD according to preoperative cerebral angiography or magnetic resonance angiography following *the Diagnostic criteria 2009 of moyamoya disease (English version published in 2012)* [5]; (b) patients were identified with intracranial hypoperfusion via preoperative magnetic resonance imaging (MRI) or positron emission tomography computed tomography examinations (PETCT), and intra- and extracranial bypass surgery was required. Exclusion criteria: (a) patients had comorbidities in the heart, liver, lungs, and kidney; (b) American society of aneshesiologistsphysical status classification system (ASA) class IV and severer; (c) pregnant or breastfeeding patients; (d) patients had incomplete clinical data or were lost to follow-up.

### Clinical data collection

We reviewed the medical records of MMD patients and categorized into the Propofol group (P group) and the Sevoflurane group (S group, Totally propofol infusion < 200 mg). Neurological endpoints were traced via a cohort study. Patients’ data were collected, including age, gender, preoperative clinical symptoms, history of hypertension, diabetes mellitus, coronary heart diseases, smoking, and drinking, body mass index (BMI index), ASA class, surgical approach, intraoperative vital signs, rehydration and urine volume, the values of hemoglobin, hematocrit, blood lactate, and blood glucose in the blood gas analysis before the end of the surgery, epilepsy incidence, hospital stay length, national institute of health stroke scale (NHISS) score at 7 days preoperatively and postoperatively and 6 months postoperatively, mRS score at 7 days preoperatively and postoperatively and 6 months postoperatively. The primary endpoint was stroke incidence within seven days after anesthesia, and the secondary endpoints included transient neurological deficits (TNDs) and incidence of postoperative Epilepsy.

We adopted average real variability (ARV) to calculate patients’ intraoperative blood pressure stability to evaluate patients’ intraoperative hemodynamic features. The formula is as follows.

ARV _BP_: =$$\:\frac{1}{\text{T}}{{\Sigma\:}}_{\text{k}=1}^{\text{N}-1}\left|{\text{B}\text{P}}_{\text{k}+1}-{\text{B}\text{P}}_{\text{k}\:}\right|{\text{m}\text{m}\text{H}}_{\text{g}}/\text{m}\text{i}\text{n}$$

Previous studies have shown that ARV more reliably estimates variability for time-series data than the standard deviation (SD) [6], and high variance of intraoperative blood pressure and drastic blood pressure decline are independent risk factors for postoperative infarction in MMD patients [7], so we calculated the ARV of blood pressure values for all patients and compared the intraoperative blood flow stability of different types of anesthetic drugs.

### MMD surgery and postoperative management

Based on Japanese MMD diagnostic and treatment guidelines, patients fulfilled the indications for surgery. The surgery was performed by a surgeon who performed over 200 operations annually from January 2019 to December 2020. Routine intraoperative indocyanine angiography showed that the bypass grafts-maintained patency without occlusion.

In our hospital’s neurosurgery department, the surgical approach for moyamoya disease is primarily determined by the patient’s intraoperative vascular conditions. The preferred method is combined surgery (middle cerebral artery-superficial temporal artery (STA-MCA) bypass combined with encephalo-duro-myosynangiosis (EDMS)). If the patient’s vascular conditions are poor, indirect surgery (encephalo-duro-arterio-myo-synangiosis (EDAMS)) is performed instead.

The anesthesiologists decides whether to use intravenous anesthetics or inhaled anesthetics to maintain anesthesia based on his clinical experience and drug usage habits, and typically follows the following protocol when administering anesthesia and perioperative management:

A. Hemodynamic ventilation management: blood pressure control mainly depends on the patient’s baseline blood pressure levels and clinical manifestations. During the surgery, the patient’s blood pressure was closely monitored invasively. SBP was strictly controlled within the normal range. Patients were transferred to NICU after they recovered from general anesthesia, and their blood pressure was measured every half hour non-invasively. During intraoperative management, the patient’s blood pressure is regulated by an experienced anesthesiologist based on preoperative measurements. Additionally, vasoactive medications such as norepinephrine or nicardipin may be utilized. Typically, blood pressure is maintained within a stable range with a slight increase of approximately 5% during the intraoperative period. An intraoperative ventilation strategy to maintain EtCO_2_ at a relatively high level (35–40) to prevent hyperventilation-induced hypocapnia, which could trigger vasospasm.
B. Infusion and management: Patients routinely had fasted at least 8 h before surgery, so they were intravenously injected with 0.9% saline or Lactated Ringer’s solution (500–1500 ml) to prevent excessive body fluid loss and volume depletion. The intraoperative infusion was contingent on hemodynamics and surgical condition. Patients were given standard rehydration volume with additional 1000–2000 ml fluids postoperatively for fluid resuscitation and blood volume increase. Mannitol was routinely applied postoperatively to prevent brain edema.
C. During the operation, fentanyl was used for anesthesia induction and remifentanil was used for analgesia maintenance. The dosage was based on the conventional anesthesia dosage. Acetaminophen for rescue analgesia in postoperative pain.

### Neurological function evaluation

We categorized the onset manifestation into 3 main types: ischemic (cerebral infarction and TIA), hemorrhagic, and nonspecific symptoms (including transient neurological deficits (TNDs) and epilepsy). All patients underwent head computed tomography (CT), magnetic resonance imaging (MRI), diffusion-weighted imaging (DWI), and digital subtraction angiography (DSA) before the surgery and immediately underwent a head CT scan after the surgery. A Head CT scan was performed 24 h postoperatively to exclude intracranial hemorrhage and/or new cerebral infarct routinely. Once novel symptoms occur, patients must promptly receive CT or MRI for further diagnosis. Novel hypointense signals on CT or hyperintense lesions and hypointense signals on DWI are considered new cerebral infarcts. Diagnosis stroke: refers to new ischemic or hemorrhagic stroke events occurring within 7 days postoperatively, confirmed by imaging examinations that identify responsible lesions. Transient neurological deficits (TNDs): Refers to new symptoms of localized neurological dysfunction occurring during the perioperative period, including aphasia, limb weakness, sensory abnormalities, epilepsy, vomiting, vertigo, facial paralysis, blurred vision, coughing while drinking water, and disturbances of consciousness. Imaging examinations reveal no new stroke-related lesions, and symptoms resolve within 7 days.

### Follow-up

According to previous studies, examinations within 30 days of surgery are defined as short-term functional evaluations. Patients were followed up via telephone, text message, and outpatient visits. Patients’ clinical outcomes were acquired, including recurrent stroke (intracranial hemorrhage or cerebral infarct after revascularization), neurological status, and radiological data. Patients were followed up via telephone or text or clinic 6 months later routinely with results recorded.

**Fig. 1:**
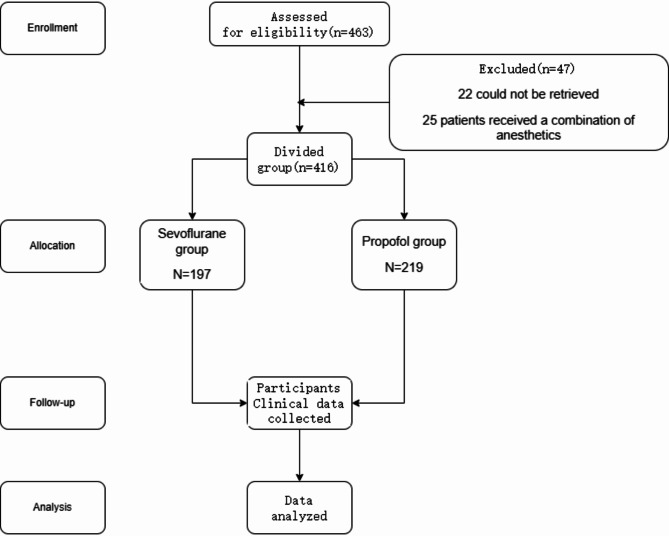
Flowchart of MMD patients included and excluded in our subject

The inclusion of Moyamoya patients in the study is shown in the flowchart below (Fig. 1).

### Statistical analysis

Statistical analysis was conducted using SPSS version 25 (IBM, New York). The methods were applied as follows: to compare categorical variables (e.g., demographic and clinical characteristics) between the inhalational anesthesia group and the intravenous anesthesia group, Fisher’s exact test was employed. For continuous variables (e.g., age, blood pressure ), the Mann-Whitney U test was used.Univariate analysis was performed to identify distinct variables between the two groups. Variables with significant differences (α < 0.05) were considered for further interpretation.A significance level of α < 0.05 was set as the threshold for statistical significance in all analyses.

## Results

### Baseline characteristics of the patients receiving inhalational versus intravenous anesthetics

Altogether 463 MMD patients were assessed in the study. 47 cases were excluded because that 22 patients’ data could not be retrieved, and 25 patients received a combination of anesthetics (given both inhalational anesthetics and persistent propofol perfusion). Hence, 197 patients who received inhalational anesthetics alone and 219 patients who received intravenous anesthetics alone were included.

The two groups were compared in Table 1. Patients from the two groups exhibited no significant differences in ASA status, history of hypertension, diabetes mellitus, and coronary heart diseases, MMD type (ischemia/hemorrhage) and preoperative neurological score at baseline.

**Table 1:** Baseline characteristics of the patients receiving Inhalational Versus Intravenous anesthetics

Characteristic	S group(197)	*P* group(219)	*P* value
**Age (median/IQR)**	46(34–52)	44(37–52)	0.438
**Male sex**,** n (%)**	90 (45.6)	107 (49)	0.556
**BMI (median/IQR)** **clinical symptoms**:	24.2 (21.9–26.2)	23.7 (21.6–25.7)	0.119
**ischemia symptoms**,** n (%)** **hemorrhagic symptoms**,** n (%)**	151 (76.7) 46(23.3)	159 (72.6) 60(27.4)	0.369
**Smoking**,** n (%)**	41 (20.8)	53 (24.2)	0.414
**Drinking**,** n (%)**	31(15.7 )	43 (19.6)	0.308
**DM**,** n (%)**	14 (7.1 )	24 (10.9)	0.233
**COPD**,** n (%)**	0 (0)	3 (1.4)	0.250
**HTN**,** n (%)**	72 (36.5)	84(38.3)	0.761
**CAD**,** n (%)**	4 (2.0)	9 (4.1)	0.268
**ASA**,** (median/IQR)**	2(2–2)	2(2–2)	0.199
**Preoperative NHISS score**,** (median/IQR)**	1(0–2)	0(0–2)	0.163
**Preoperative mRS score**,** (median/IQR)**	1(1–2)	1(1–2)	0.572
**Type of surgery**,** combined**,** n %**	182(92.4)	193(88.1)	0.243

Categorical variables are represented as number (percent). Continuous variables are presented as presented as median/IQR.BMI, body mass index; DM, diabetes mellitus; COPD, chronic obstructive pulmonary disorder; HTN, hypertension; CAD, coronary artery disease; ASA, American Society of Anesthesiology; NHISS, National Institute of Health stroke scale; mRS, Modified Rankin Rating Scale; IQR, interquartile range. *P* < 0.05 is statistically significant

### Intraoperative hemodynamic and postoperative characteristics of patients receiving inhalational anesthesia versus intravenous anesthesia

All patients included in the study were collected for intraoperative blood pressure data, end-expiratory carbon dioxide, and the last blood gas analysis value during surgery. We analyzed all blood pressure data, including the average values of systolic blood pressure (SBP), diastolic blood pressure (DBP), and mean blood pressure (MBP), as well as each blood pressure ARV values of each patient, and compared them.

Our research results found that under standardized blood pressure control, there was no significant difference between the average systolic blood pressure of 129mmHg in group S and the average systolic blood pressure of 127 in group P (*p* = 0.248), and there was no significant statistical difference between the diastolic blood pressure of 74 in group S and 75 in group P (*p* = 0.131). However, there was a difference in MBP between the two groups, with MBP in group S being 110 compared to MBP in group P being 95 (*p* < 0.001), and there were differences in SBP, DBP, and MBP variability between the two groups, ARV_SBP_ 6.4 in group S was higher than ARV_SBP_ 5.2 in group P (*p* < 0.001). ARV_DBP_ 3.9 in group S was higher than ARV_DBP_ 3.2 in group P (*p* = 0.002), and ARV_MBP_ 4.5 in group S was higher than ARV_MBP_ 3.8 in group P (*p* = 0.001). These indicators indicate that the hemodynamic stability in the propofol group is higher than that in the sevoflurane anesthesia group. In terms of blood gas indicators, there was no significant statistical difference between the two.

**Table 2:** Intraoperative hemodynamic and blood gas analysis characteristics of patients receiving inhalational anesthesia versus intravenous anesthesia

Characteristic	S group(197)	*P* group(219)	*P* value
**Average SBP (median/IQR)**	129(120–138)	127(119–137)	0.248
**ARV SBP (median/IQR)**	6.4(4.7–8.2)	5.2(3.2–7.2)	< 0.001^*^
**Average DBP (median/IQR)**	74(68–79)	75(68–81)	0.131
**ARV DBP (median/IQR)**	3.9(2.9-5.0)	3.3(2.1–4.7)	0.002^*^
**Average MBP (median/IQR)**	110(102–117)	95(89–103)	< 0.001^*^
**ARV MBP (median/IQR)**	4.5(3.3-6.0)	3.8(2.4–5.5)	0.001^*^
**Average EtCO**_**2**_ **(mmHg)(median/IQR)**	36.3(34.2–38.0)	36.6(34.1–40.0)	0.032^*^
**HB (g/L) (median/IQR)**	119(108–130)	119(107–129)	0.711
**HCT (%) (median/IQR)**	35(32–38)	36(32–40)	0.081
**LAC (mmol/L) (median/IQR)**	1.2(0.9–1.7)	1.3(1.0-1.9)	0.110
**GLU (mmol/L) (median/IQR)**	5.5(5-6.2)	5.4(4.8–6.7)	0.534

Categorical variables are represented as number (percent). Continuous variables are presented as presented as median/IQR.SBP, systolic blood pressure; DBP, diastolic blood pressure; MBP, mean blood pressure; EtCO_2_, end-expiratory carbon dioxide; Hb, Hemoglobin; HCT, Red blood cell specific volume; LAC, Blood Lactic Acid; GLU, Blood glucose; ^*^P < 0.05 is statistically significant

### Patient hospitalization days indicators, postoperative neurological outcomes, and postoperative neurological function scores

In our study, there was no statistical difference in the total length of hospitalization between different groups (*p* = 0.354), and there was a statistical difference in the length of ICU stay. However, the p_25_, p_50_, and p_75_ values were all one day, and the overall difference was not significant. There was no significant difference in stroke incidence between the two groups (S group vs. P group = 6.6% vs. 5.9%, OR = 0.893; 95% CI, 0.404–1.976; *p* = 0.780). There was no statistical difference between the two groups in the occurrence of postoperative TNDs(S group vs. P group = 32.5% vs. 31.1%, OR = 0.936; 95% CI, 0.619 ~ 1.415, *p* = 0.753) and epilepsy (S group vs. P group = 5.1% vs. 5.0%, OR = 0.989; 95% CI, 0.411 ~ 2.328, *p* = 0.980). There were statistical differences in the NHISS score (S group vs. P group = 2:1, *p* < 0.001) and mRS score (S group vs. P group = 2:1, *p* < 0.001) at 7 days after surgery, as well as the mRS score at 6 months of follow-up (S group vs. P group = 0:0, *p* < 0.001), but the difference in scores was small. but when compared mRS score p_75_ (S group vs. P group = 3:1) in 6 month was quite different in each group.

We included the factors with statistically significant differences into the regression equation for analysis (Table 4). Multivariate regression analysis showed that the choice of anesthetic drugs affected the postoperative mRS score, intraoperative MBP level, and ARV DBP.

**Table 3:** Patient hospitalization days indicators, postoperative neurological outcomes, and postoperative neurological function scores

Characteristic	S group(197)	*P* group(219)	*P* value
**ICU length of stay (median/IQR)**	1(1–1)	1(1–1)	0.008^*^
**Total hospital length of stay (median/IQR)**	14(12–16)	14(13–17)	0.354
**TNDs** **n (%)**	64(32.5)	68(31.1)	0.753
**Stroke** **n (%)** **cerebral infarctionn (%)** **cerebral hemorrhagen (%**)	13(6.6) 11(5.6) 2(1.0)	13(5.9) 10(4.6) 3(1.4)	0.780 0.636 1.000
**Epilepsy** **n (%)**	10(5.1)	11(5.0)	0.980
**NHISS score in 7 days (median/IQR)**	2(0–4)	1(0–4)	0.082
**mRS score in 7 days (median/IQR)**	2(0–3)	1(0–2)	< 0.001
**mRS score in 6 Month (median/IQR)**	0(0–3)	0(0–1)	< 0.001

Categorical variables are represented as number (percent). Continuous variables are presented as presented as median/IQR. ICU, intensive care of unit; TNDs, transient neurological deficits; *P* < 0.05 is statistically significant

**Table 4:** Multivariate logistic regression analysis of influence factors of anesthetics

Variables	β	S.E	Z	*P*	OR (95%CI)
**mRS score in 7 days**	1.533	0.419	3.662	< 0.001^*^	4.633 (2.039 ~ 10.525)
**mRS score in 6 months**	-1.851	0.414	-4.471	< 0.001^*^	0.157 (0.070 ~ 0.354)
**MBP**	-0.121	0.015	-8.361	< 0.001^*^	0.886 (0.861 ~ 0.911)
**ARV SBP**	-0.109	0.105	-1.043	0.297	0.896 (0.730 ~ 1.101)
**ARV MBP**	0.192	0.171	1.128	0.259	1.212 (0.868 ~ 1.694)
**ARV DBP**	-0.301	0.148	-2.037	0.042^*^	0.740 (0.554 ~ 0.989)
**EtCO**_**2**_	-0.049	0.042	-1.181	0.237	0.952 (0.877 ~ 1.033)
**ICU length of stay**	0.057	0.111	0.511	0.609	1.059 (0.851 ~ 1.317)

OR: Odds Ratio, CI: Confidence Interval, mRS, Modified Rankin Rating Scale.SBP, systolic blood pressure; DBP, diastolic blood pressure; MBP, mean blood pressure. EtCO_2_, end-expiratory carbon dioxide *.P* < 0.05 is statistically significant. ^*^P < 0.05 is statistically significant

## Discussion

Previous studies revealed that conservative therapies for MMD patients had poor efficacy, were only symptomatic treatments, and could not arrest disease progression [8]. The primary surgical approach of MMD is surgical revascularization. After analyzing patients with moyamoya disease who underwent bypass surgery with different types of anesthetic agents, we reached the following findings: (1) There was no clear effect of the choice of anesthetic agent on the primary or secondary outcomes in MMD patients who received either intravenous propofol or inhalational anesthetics for general anesthesia, specifically regarding TNDs (including epilepsy) and short-term stroke following surgical revascularization. (2) Intravenous anesthetics provided patients with better hemodynamics compared with inhalational anesthetics during MMD surgery (ARV_MBP_: 4.5 vs. 3.8, *p* < 0.001, ARV_SBP_: 6.4 vs. 5.2, *p* < 0.001, ARV_DBP_: 3.9 vs. 3.3, *p* < 0.001).

Multiple studies revealed that inhalational anesthetics protect patients with cerebrovascular diseases and neurovascular defects. Sevoflurane pretreatment can reduce vasospasm of large arteries and microthrombosis to improve neurological functions [9]. In propofol-treated rat models with ischemic brain injury, the degree of brain edema was reduced with infarct size reduced [8]. Besides, the expression of inflammatory factors, TNF-α and IL-1β was reduced with upregulated phosphorylated Akt expression. Following the administration of Akt inhibitor LY294002, propofol’s anti-infective effects and activities in upregulating Akt expression were suppressed. In subarachnoid hemorrhages and relevant early brain injury, propofol can inhibit inflammation via the PI3K/Akt pathway, significantly improve blood-brain barrier permeability, neurological dysfunction, and brain edema to protect neurons [10]. Currently, the primary treatment for MMD patients is surgery owing to the unsatisfactory efficacy of conservative treatment. The principal surgical approach is combined surgery (middle cerebral artery (STA-MCA) bypass + encephalo-duro-myosinangiosis (EDMS)) or indirect surgery (encephalo-duro-arterio-myo-synangiosis (EDAMS)) [11], The primary goal of surgery is to improve internal carotid-external carotid (IC-EC) conversion [12] to prevent further neurological and cognitive function impairment due to MMD development. Previous studies demonstrated that different surgical approaches presented no significant differences in postoperative ischemic events [13].

Blood pressure control is an essential aspect of MMD perioperative treatment. Fujimura M et al. [14] reported that preventive blood pressure reduction was conducive to preventing symptomatic brain hyperperfusion, in which symptomatic brain hyperperfusion in patients was improved without inducing permanent neurological deficits. The P group had a lower intraoperative MAP than the S group. Different characteristic changes in blood flow caused by anesthetic agents may contribute to different postoperative mRS scores among MMD patients, possibly affecting patients’ prognoses. When it comes to hemodynamic stability, the choice between inhalation anesthesia and intravenous anesthesia can have a significant impact on the surgical process and postoperative recovery of Moyamoya disease patients. Inhalation anesthesia and intravenous anesthesia have different effects on hemodynamics. Inhalation anesthesia can control the depth and frequency of anesthesia by adjusting breathing, and it can provide more stable hemodynamics. However, inhalation anesthesia may also cause vasodilation and reduce blood pressure, which could adversely affect cerebral perfusion in Moyamoya disease patients. In contrast, intravenous anesthesia typically allows more precise control of the level of anesthesia, as it can be gradually adjusted based on the patient’s specific condition. Additionally, intravenous anesthesia can quickly adjust hemodynamic parameters by modifying the infusion rate. This makes intravenous anesthesia more suitable when precise hemodynamic control is needed, such as for patients with cardiovascular diseases.

TNDs is a prevalent complication after MMD surgery [15]. Uno et al. first identified the phenomenon of transient neurological deficits (TND) associated with hyperperfusion on SPECT imaging in patients with moyamoya disease (MMD) [16]. Usually, patients don’t exhibit significant ischemia or hemorrhage during imaging examinations but present certain neurological disorders (limb function, sensation, aphasia, etc.). Most neurological disorders will improve or disappear before discharge. Regarding anesthetic agent selection in our study, propofol and sevoflurane showed no significant differences in postoperative TNDs incidence. However, it is important to consider other crucial factors that contribute to the occurrence of TNDs, such as surgical vessel selection and the patient’s acceptance of graft vessels. These factors play a critical role in determining the incidence of TNDs in MMD surgery. Mukerji et al. further hypothesized that TNDs could result from impaired cerebral blood flow autoregulation and local hypoperfusion due to competitive blood flow [17]. Additionally, Phi et al. proposed an alternative explanation, suggesting that TND after indirect bypass may be attributed to transient cortical depression triggered by mechanical stimulation rather than hyperperfusion [18]. It is essential for doctors to carefully monitor patients undergoing MMD surgery for any signs of TNDs and take appropriate measures to manage and mitigate its effects. We hypothesize that more stable intraoperative hemodynamics may reduce the occurrence of postoperative TNDs. In the univariate analysis, the ARV of the propofol group was lower compared to the sevoflurane group, indicating less blood pressure fluctuation. Furthermore, after multivariate analysis, the ARV of MBP remained lower in the propofol group, suggesting that propofol offers more competitive hemodynamic management during surgery, which may help reduce the incidence of postoperative TNDs.

In summary, although our research suggests that there is no difference in the incidence of postoperative neurological complications among different anesthetic drugs, choosing the appropriate anesthetic drugs to maintain good hemodynamic stability is crucial for bypass surgery in Moyamoya disease patients. Anesthesiologists will select the most suitable anesthetic drugs based on the patient’s specific condition and surgical requirements, closely monitoring and adjusting hemodynamic parameters to ensure the safety and success of the surgical process.

## Limitation

There are some limitations to this study. First, this study is a non-randomized retrospective study, the anesthesiologist decides whether to use intravenous anesthetics or inhaled anesthetics to maintain anesthesia based on his clinical experience and drug usage habits will take possible selection bias. Second, all data comes from the same research center, and there may be some bias in sample selection. Thirdly, the observation of postoperative complications in this study did not consider the influence of other factors such as vasoactive drugs after anesthesia. Fourthly, we didn’t have access to unconventional monitoring data such as intraoperative brain oxygen content. Hence, we expect to further explore risk factors during MMD surgery and reduce complication incidence via more multi-center prospective randomized controlled studies.

## Conclusion

In general, the effects of inhalation anesthesia and intravenous anesthesia in moyamoya disease bypass surgery may involve hemodynamic stability, cerebral blood flow, and postoperative neurologic function recovery. MMD patients who received inhalational anesthetics and intravenous anesthetics had similar odds of neurological deficits. During the operation hemodynamic stability in the propofol group is greater than that in the sevoflurane anesthesia group suggesting that propofol offers more competitive hemodynamic management during surgery. Choosing the appropriate anesthesia drugs and individualized anesthesia management by a professional anesthesiologist based on the specific condition of the patient is crucial for the success of the surgery and postoperative prognosis.

## Data Availability

The database is private and there is no public access and our data include identifiable human data and might have a risk of reidentification if shared openly. However, permission to access and use the database can be obtained if necessary, by request to the corresponding author.
